# Effects of post-stress corticosterone on hippocampal excitability and behavior involving hyperpolarization-activated cation channel 1 function

**DOI:** 10.21203/rs.3.rs-7014211/v1

**Published:** 2025-07-11

**Authors:** Chung Sub Kim, Jiwon Kim, Sandali Michael

**Affiliations:** Augusta University

**Keywords:** Contextual fear conditioning, corticosterone, fear memory, dorsal CA1 neurons, neuronal excitability, h current

## Abstract

Single Prolonged Stress (SPS) is a widely used animal model for investigating the physiological and behavioral consequences of acute stress exposure. Glucocorticoids released during stress can induce atypical fear memories, including contextual amnesia and emotional hypermnesia. Hyperpolarization-activated cyclic nucleotide-gated 1 (HCN1) channels are abundantly expressed in the dorsal CA1 (dCA1) region of the hippocampus, where they influence both intrinsic neuronal excitability and synaptic function. Although we have previously shown that acute corticosterone (CORT) exposure increases hyperpolarization-activated current (*I*_h_) in dCA1 neurons *in vitro*, it remains unclear whether *in vivo* CORT exposure following stress exerts similar effects and contributes to behavioral dysfunction. To address this, 8–9-week-old male mice were subjected to SPS followed by treatment with either vehicle or CORT. Behavioral assays—including the open field test, Y-maze, and contextual fear conditioning—were conducted, followed by whole-cell patch-clamp recordings in dCA1 neurons. Mice treated with SPS and post-CORT exhibited deficits in spatial working memory, contextual recall, and fear extinction, mimicking PTSD-like symptoms. Electrophysiological recordings revealed that dCA1 neurons from these mice displayed decreased input resistance, reduced action potential firing, and elevated *I*_h_. These alterations were reversed by ZD7288, an HCN channel blocker. Moreover, overexpression of *HCN1* in dCA1 neurons in SPS-treated mice reproduced both the behavioral and physiological phenotypes observed in the SPS-CORT group. Collectively, these findings suggest that post-stress CORT exposure promotes maladaptive hippocampal plasticity via enhanced HCN1 activity, linking stress hormones to altered hippocampal function and PTSD-like behavioral outcomes.

## Introduction

After being exposed to traumatic events, people can develop post-traumatic stress disorder (PTSD), a crippling mental illness marked by heightened arousal, avoidance, negative changes in mood and cognition, and persistent re-experiencing^1^. There is evidence of hippocampal atrophy and impaired memory processing in those who have PTSD, and an increasing amount of neuroimaging and clinical research has linked dysfunction in the hippocampus, amygdala, and prefrontal cortex as key neural substrates of the disorder^2–4^ Rodent models, like the single prolonged stress (SPS) paradigm, have been created to close the translational gap^5, 6^ SPS was first shown to produce PTSD-like symptoms in rats by combining restraint, forced swimming, and ether exposure^6^. These symptoms include increased anxiety, poor fear extinction, and dysregulated HPA-axis function^5–7^. These behavioral effects have not always been replicated in mice, though^8, 9^. Despite evidence of neuroinflammation and decreased neurogenesis, recent studies using mice show no discernible changes in traditional tests such as the elevated plus maze, Y-maze, or open field test^8, 9^. This implies that SPS may induce a latent neural vulnerability in mice that needs a second challenge to become behaviorally apparent. Elevated glucocorticoids, like corticosterone (CORT), can significantly impair hippocampus memory processing when under stress^10, 11^. Contextual amnesia results from direct glucocorticoid infusion into the dorsal CA1 region, which also enhances emotional hypermnesia, or the heightened recall of trauma-associated cues^12, 13^. These results show that dorsal CA1 dysfunction is causally linked to the development and maintenance of maladaptive fear memories, and that hippocampal glucocorticoid signaling also plays a key role in PTSD-like memory disorders. In our previous study, we demonstrated that acute corticosterone treatment *in vitro* significantly increases hyperpolarization-activated cation channel 1 (HCN1) protein expression in both the somatic and distal dendritic compartments of dorsal CA1 pyramidal neurons, leading to an increase in membrane-bound h current (*I*_h_)^14^. These effects of corticosterone are mediated by Glucocorticosteroid Receptor (GR)-HCN channels-Protein Kinase A (PKA) pathway^14^. HCN1 channels have also been implicated in PTSD-like and depression-related phenotypes^15–18^. In rodent models such as the chronic unpredictable stress paradigm in rats and the chronic social defeat stress model in mice, HCN1 modulation influences not only neuronal excitability, but also key molecular markers including brain-derived neurotrophic factor and mammalian target of rapamycin, as well as synaptic transmission and behavioral responses to stress and trauma^14, 16, 17, 19, 20^. Given (1) the inconsistent behavioral effects of SPS in mice and (2) the unclear impact of *in vivo* corticosterone on *I*_h_, we administered corticosterone immediately following the SPS paradigm. This approach aligns with the framework of “two-hit” stress models, in which an initial traumatic event primes the neural system, and a subsequent hormonal or environmental challenge reveals latent PTSD-like impairments^21^. In our model, SPS with post-CORT administration produced marked deficits in spatial working memory, fear memory retrieval, and fear extinction, whereas SPS with post-vehicle had no significant behavioral effects. In addition, dorsal CA1 neurons from SPS-CORT mice exhibited decreased input resistance, reduced action potential firing, and increased *I*_h_, indicating altered intrinsic excitability, whereas changes were not observed in the SPS-Veh group. Finally, SPS-treated mice with *HCN1* overexpression displayed behavioral deficits similar to those observed in SPS-CORT mice, supporting a key role for HCN1 channels in mediating stress-induced behavioral and physiological dysfunction. Our results demonstrate that post-traumatic glucocorticoid exposure unmasks latent neural vulnerabilities established by prior stress and that HCN1-mediated enhancement of *I*_h_ in dorsal CA1 neurons may serve as a mechanistic link between stress sensitization and PTSD-like symptoms.

## Materials and Methods

### Animals

Male C57BL/6J mice aged 7 to 10 weeks were used in this study. Mice were housed 2–5 per cage under a 12-hr light/dark cycle (lights on at 6 am, off at 6 pm) with *ad libitum* access to water and food. All procedures involving animals were approved by the Institutional Animal Care and Use Committee of Augusta University.

### Drugs

ZD7288 (Cat #1000) was obtained from TOCRIS. (2-Hydroxypropyl)-β-cyclodextrin (HBC; Cat# H107) and Corticosterone-HBC complex (Cat# C174) were obtained from Sigma-Aldrich.

### Viruses

pLenti-CaMKIIα-GFP (#VB220317–1266jgr) and pLenti-CaMKIIα-HCN1-GFP (#VB220317–1258jak) were purchased from VectorBuilder (USA).

### Single prolonged stress procedures

The single prolonged stress (SPS) protocol consists of three sequential stressors: 2-hr restraint, 15-min forced swim, and exposure to isoflurane anesthesia (2%). Briefly, mice were restrained for 2 hr in a small acrylic tube (50 ml). Immediately afterward, the mice underwent a 15-min forced swim stress in a transparent Plexiglass cylinder (25 cm tall × 20 cm in diameter) filled with water (23°C – 24°C) to a depth of 15 cm. Following the swim, mice were dried by a towel and exposed to 2% isoflurane for 5 min using a soaked cotton pad in a closed plastic container. After SPS, mice were singly housed and left undisturbed for 10 days.

### Behavioral experiment

The behavioral experiment was conducted under blind conditions between 4 pm and 6 pm.

### Open field test

Anxiety-like behavior and locomotor activity were assessed using the open field test as described previously^17^. Each mouse was placed in the center of the open field arena and allowed to explore freely for 6 minutes while being recorded by a CCD camera. The arena was cleaned with 70% ethanol between trials to eliminate odor cues. Key parameters measured included total distance traveled, number of entries into the center zone, and the duration spent in the center. A line crossing was counted when the mouse’s body center crossing a demarcation line.

### Y-maze spontaneous alternation test

Short-term spatial working memory was evaluated with the Y-maze spontaneous alternation test, as described previously^14, 17^. The maze consists of three identical arms (14 in × 3 in) arranged at 120° angles without intra-maze cues or arm blocking. Mice were placed in one arm and allowed to explore for 6 minutes. Arm entries (defined by all four paws entering an arm) and sequences were recorded via overhead CCD camera. Alternation percentage was calculated as the ratio of actual alternations to the maximum possible (total entries minus two). Additional metrics included total arm entries and self-arm return (SAR). The maze was cleaned with 70% ethanol between animals.

### Contextual fear conditioning, fear memory, and fear extinction

To assess fear memory and extinction, a tone-shock unpairing protocol was used as described previously^12, 17^. On the first day of the protocol, referred to as the habituation phase (Day 1), each mouse was individually introduced into a specific testing environment designated as context B. This chamber was constructed with an opaque polyvinyl chloride (PVC) floor designed to minimize visual cues from below, thereby providing a unique sensory environment distinct from the conditioning context. The animals were allowed to freely explore this chamber for a total of 4 minutes to acclimate to the novel surroundings. To ensure consistency and eliminate residual olfactory cues that could influence behavior, the chamber was carefully cleaned with a 1% acetic acid solution prior to the introduction of each mouse. The following day, the fear conditioning acquisition session (Day 2) was conducted in a separate and distinct environment, context A, chosen to provide a different set of sensory cues and spatial configuration to the animals. Context A consisted of a square chamber measuring 24 by 24 centimeters, featuring a floor composed of nineteen stainless steel rods spaced 1 centimeter apart and capable of delivering controlled electrical foot shocks. The shock intensity was set to 0.6 milliamperes to reliably induce a fear response without causing undue distress. Prior to each trial, this chamber was sanitized thoroughly with 70% ethanol to prevent carryover of scent cues. During the conditioning session, mice were placed into context A and given 100 seconds to acclimate before any stimuli were presented. Subsequently, an auditory tone with a frequency of 1 kHz and intensity of 65 dB was delivered for 15 seconds. A foot shock lasting one second was administered during the final second of the tone presentation. This tone-shock pairing was presented in an unpaired manner, with specific intervals separating the stimuli to prevent temporal association. 24 hours later (Day 3), memory retrieval was assessed through two separate tests. First, mice were returned to context B for a tone re-exposure test lasting 6 minutes. This session was divided into three 2-minute phases: pre-tone, tone presentation, and post-tone. Freezing behavior, defined as the absence of all movement except respiration, was quantified during each phase using FreezeView 5 software (Actimetrics, USA). The freezing data were then used to calculate a tone ratio reflecting conditioned fear responses while controlling for baseline activity. 2 hours after the tone test, mice underwent a context re-exposure test in context A for 6 minutes, where freezing behavior was again monitored to assess memory of the conditioning environment. Fear extinction was evaluated in subsequent sessions where mice were placed in context A for 6 minutes daily across three consecutive days. Freezing behavior during these sessions was tracked to measure the reduction of conditioned fear responses over time. Throughout all behavioral testing phases, continuous white noise at 55 to 60 dB was maintained to mask any extraneous environmental sounds, thereby minimizing confounding auditory stimuli and helping to ensure that behavioral responses were specific to the experimental manipulations. The tone ratio is calculated as follows: [% freezing during tone presentation−(% pre-tone period freezing+% post-tone period freezing)/2]/[% freezing during tone presentation+(% pre-tone period freezing+% post-tone period freezing)/2]^12, 17^ All experiments were conducted with investigators blinded to treatment groups and during the time window of 10 am to 2 pm to reduce variability related to circadian influences.

### Stereotaxic microinjection

Stereotaxic microinjections were carried out following established protocols with minor modifications^17, 22^ Prior to surgery, animals received a subcutaneous injection of carprofen (5 mg/kg, Covetrus). Male C57BL/6J mice aged 5 weeks were anesthetized with 0.5 L/min oxygen and 2% isoflurane throughout the procedure. Using a Hamilton syringe with a 26s-gauge needle (Hamilton, USA), mice were bilaterally injected with either pLenti-CaMKIIα-GFP or pLenti-CaMKIIα-HCN1-GFP into the dorsal hippocampal CA1 region (−1.8 mm anterior-posterior, ± 1.6 mm medial-lateral, −1.4 mm dorsal-ventral from the dura) for the overexpression of the *HCN1* gene. The injected volume was 0.4 μl per hemisphere, delivered slowly over 5 minutes. Following infusion, the injection needle was maintained in place for at least an additional 5 minutes to allow for diffusion and prevent backflow. Following the stereotaxic injection, the mice were returned to their home cages, and their body weight was monitored regularly to assess recovery and well-being.

### Acute hippocampal slice preparation

Mice were anesthetized with isoflurane gas (2–3%) in a closed plastic container and transcardially perfused with ice-cold artificial cerebral spinal fluid (aCSF) composed of (in mM): 2.5 KCl, 1.25 NaH_2_PO_4_, 25 NaHCO_3_, 0.5 CaCl_2_, 7 MgCl_2_, 7 dextrose, 210 sucrose, 1.3 ascorbic acid, and 3 sodium pyruvate, bubbled with 95% O_2_ − 5% CO_2_. The brain was removed and hemisected along the longitudinal fissure. Dorsal hippocampal slices were prepared as previously described^14, 17^ 300 μm thick hippocampal slices were made in ice-cold aCSF using a vibrating microtome (Microslicer DTK-Zero1, DSK, Kyoto, Japan). Slices were placed in a holding chamber containing (in mM) 125 NaCl, 2.5 KCl, 1.25 NaH_2_PO_4_, 25 NaHCO_3_, 2 CaCl_2_, 2 MgCl_2_, 12.5 dextrose, 1.3 ascorbic acid, and 3 sodium pyruvate, bubbled with 95% O_2_ − 5% CO_2_ at 35°C for 30 min and then incubated for at least 45 min at room temperature before used for electrophysiology. Whole-cell patch-clamp recordings were performed as previously described^14, 17^. Briefly, hippocampal slices were submerged in a recording chamber continuously perfused with aCSF containing (in mM) 125 NaCl, 3 KCl, 1.25 NaH_2_PO_4_, 25 NaHCO_3_, 2 CaCl_2_, 1 MgCl_2_, and 12.5 dextrose, bubbled with 95% O_2_ − 5% CO_2_ at a rate of 1 ml/min and 31–33°C. CA1 pyramidal neurons were visually identified using a microscope (Olympus BX51WI, US) fitted with differential interference contrast optics^23^. Patch pipettes for somatic (4–7 MΩ) were prepared with capillary glass (external diameter 1.65 mm and internal diameter 1.1 mm, World Precision Instruments) using a Flaming/Brown micropipette puller (P-1000, Sutter Instrument, CA) and filled with an internal solution containing (in mM) 120 K-gluconate, 20 KCl, 10 HEPES, 4 NaCl, 7 K2-phosphocreatine, 4 Mg-ATP, 0.3 Na-GTP (pH 7.3 with KOH). Whole-cell patch-clamp recordings were conducted using a MultiClamp 700B amplifier (Molecular Devices, LLC., CA) and acquired with pCLAMP10 software (Molecular Devices, LLC., CA). Electrical signals were filtered at 10 kHz, sampled at 20 kHz, digitized by Axon Digidata 1440A (Axon Instruments), and analyzed offline. For whole-cell current-clamp recordings, series resistance was continuously monitored; recordings were excluded if the series resistance exceeded 15 MΩ during the recordings. Resting membrane potential was defined as the membrane potential recorded in the absence of injected current. Liquid junction potential was not corrected but was estimated to be approximately – 13 mV using the Patcher’s Power Tools plugin in Igor Pro.

### Immunohistochemistry

Immunohistochemistry was carried out as described previously^14, 17^. Viral-infected dorsal hippocampal slices (80 μm thick) were prepared using a freezing microtome and stored in a cryoprotectant solution containing 30% sucrose, 30% ethylene glycol, 1% polyvinyl pyrrolidone, 0.05 M sodium phosphate buffer for immunohistochemistry. Sections were briefly rinsed in PBS buffer and incubated in 0.1% TritonX-100 for 30 min. Subsequently, slices were blocked in PBS solution containing 5% normal goat serum, 0.03% TritonX-100 for 1 hr, and then incubated in primary antibody diluted in blocking solution overnight at 4°C. Slices were rinsed in PBS buffer and then incubated in secondary antibody for 1hr at room temperature. Primary antibody in this study was used as follow; rabbit-anti-HCN1 (1:500, Invitrogen, Cat # PA5–78675).

## Data Analysis

Input resistance was measured by the slope of the linear fit of the V-I plot between + 30 and − 150 pA current injections. Electrophysiological data were analyzed in Easy Electrophysiology and Axograph.

## Statistical Analysis

Statistical comparisons were performed using One-Way ANOVA or the Kruskal-Wallis test followed by Dunn’s multiple comparisons test, and Two-Way ANOVA followed by Tukey post-hoc tests or Sidak’s multiple comparisons test, paired t-test (Wilcoxon signed-rank test), or unpaired t-test (Mann-Whitney U test) using GraphPad software. Statistical significance was considered at *P < 0.05, ^#^P < 0.05, and ^$^P < 0.05. For a detailed description of the statistical analyses and methods, please refer to the Supplemental Information.

## Results

### SPS-CORT mice display impaired short-term working memory, contextual amnesia, and impaired fear extinction.

**Figure 1:**
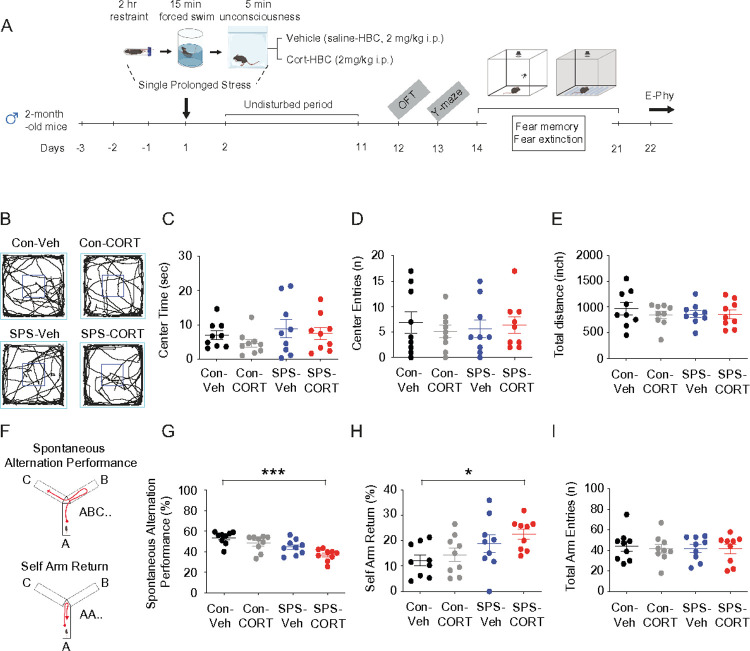
SPS-CORT mice exhibit impaired short-term spatial working memory. (A) Schematic diagram of the experimental design for single prolonged stress, behavioral tests, and electrophysiology. (B) Representative video tracking images of age-matched male mice during open filed test. (C) Center time. (D) Center entries. (E) Total travelled distance. (F) Illustration of the spontaneous alternation Y maze test. (G) Percentage of spontaneous alternation performance among groups. (H) Percentage of self-arm return. (I) Total arm entries. The Kruskal-Wallis test was performed followed by Dunn’s multiple comparisons test in (G) and (H). Data are expressed as mean ± SEM. *P<0.05 and ***P<0.001. Further statistical information is provided in the Supplemental Information. Panel A was created with BioRender.com.

**Figure 2:**
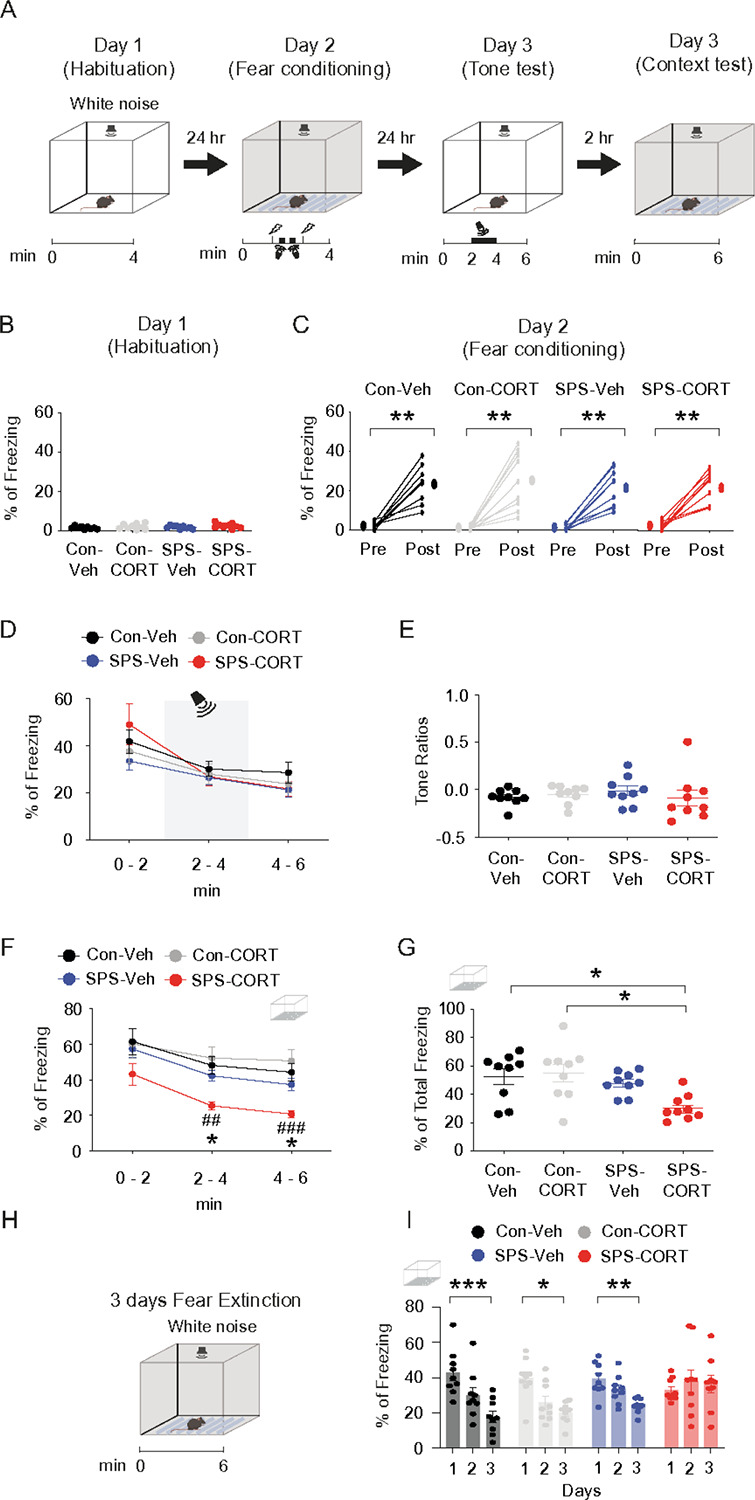
SPS-CORT mice exhibit contextual amnesia and impaired fear extinction. **(A)** Illustration showing the contextual fear conditioning with the CS-US unpairing protocol in control or SPS mice with Veh or CORT treatment. (B) Percentage of freezing during the habituation phase. (C) Fear acquisition on Day 2 was measured both before (first 1 min) and after (last 1 min) the session. The Wilcoxon matched-pairs signed rank test was performed separately for the control-Veh, control-CORT, SPS-Veh, and SPS-CORT groups. (D) Percentage of freezing during the tone memory test. The tone was present during the 2–4-minute session of the experiment. (E) Tone ratios. (F) Percentage of freezing during the context memory test. The Two-Way ANOVA followed by Tukey’s post-hoc test was performed. * indicates a comparison between the control-Veh and the SPS-CORT group. ^#^ indicates a comparison between the control-CORT and the SPS-CORT group. (G) The total percentage of freezing during a 6-minute context memory test. (H) Illustration of fear extinction experiment. (I) Fear extinction during the 3-day fear extinction test. The mouse was placed into the context for 6 minutes per day for 3 consecutive days. The Kruskal-Wallis test was performed followed by Dunn’s multiple comparisons test separately for the control-Veh, control-CORT, SPS-Veh, and SPS-CORT groups. Data are expressed as mean ± SEM. *P<0.05, **P<0.01, ***P<0.001, ^##^P<0.01, and ^###^P<0.001. Further statistical information is provided in the Supplemental Information. Panel A and H were created with BioRender.com.

Two-month-old male mice were subjected to the SPS procedure, while age-matched controls remained unexposed (Fig. 1A). Given that acute corticosterone treatment has been shown to upregulate HCN1 protein expression and enhance *I*_h_ *in vitro*^14^, we asked whether post-SPS corticosterone administration would influence behavioral outcomes. Mice received either vehicle (saline-HBC; 2 mg/kg, intraperitoneal [i.p.]) or corticosterone (CORT-HBC; 2 mg/kg, i.p.) immediately following the SPS procedure (Fig. 1A). In rodent models, particularly in rats, a recovery period of at least 7 days is considered critical for the emergence of PTSD-like phenotypes, including heightened anxiety, depressive-like behavior, hyperarousal, social withdrawal, impaired fear extinction and cognition, and HPA axis dysregulation^24^. Following a 10-day undisturbed period, we performed a battery of behavioral assessments. In the open field test (Fig. 1B), no significant group differences were observed in center time (Fig. 1C), center entries (Fig. 1D), or total distance traveled (Fig. 1E), indicating no gross locomotor or anxiety-like alterations. However, in the Y-maze test (Fig. 1F), SPS-CORT mice exhibited significantly reduced spontaneous alternation performance (SAP; Fig. 1G) and increased same-arm returns (SAR; Fig. 1H), while the total number of arm entries remained unchanged (Fig. 1I), suggesting deficits in spatial working memory rather than changes in exploratory activity. We next assessed fear memory and extinction using a contextual fear conditioning paradigm with an unpaired presentation of the conditioned stimulus (CS) and unconditioned stimulus (US) (Fig. 2A). In this protocol, the context served as the CS and the foot shock as the US, while the tone was presented in an unpaired manner and did not predict the shock. As a result, the context became the primary predictor of the aversive event, driving the freezing response^12, 17^. No significant differences were observed among the groups during habituation on Day 1 or fear acquisition on Day 2. On Day 3, mice underwent a tone re-exposure test to evaluate tone-associated freezing responses. As anticipated under the CS-US unpairing protocol, the tone did not serve as a predictive cue, resulting in no significant increase in freezing during tone presentation (Fig. 2D). Consistently, there were no group differences in freezing behavior during the tone test (Fig. 2D) or in tone discrimination ratios (Fig. 2E). Two hours later, mice were re-exposed to the original conditioning context in which they had previously received foot shocks. Notably, SPS-CORT mice displayed significantly reduced freezing during the context test (Fig. 2F), along with a decrease in the percentage of total freezing duration (Fig. 2G), indicative of contextual amnesia. Finally, we assessed fear extinction across three consecutive days of context re-exposure. Control-Veh, Control-CORT, and SPS-Veh mice exhibited a significant reduction in freezing behavior by Day 3 relative to Day 1, indicating successful extinction. In contrast, SPS-CORT mice failed to show this reduction, suggesting impaired fear extinction.

### Dorsal CA1 neurons from SPS-CORT group exhibit decreased input resistance, reduced action potential firing, and increased I_h_.

**Figure 3:**
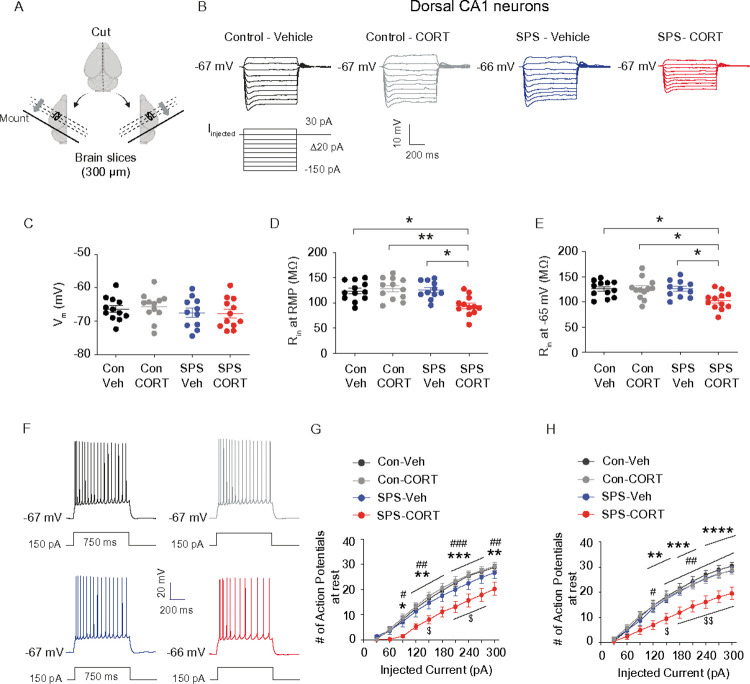
Dorsal CA1 neurons from SPS-CORT group exhibit decreased input resistance and reduced action potential firing. (A) Illustration of the preparation of dorsal hippocampal slices. (B) Representative voltage responses to a current step (−150 pA to 30 pA, D10 pA, 700 ms) at resting membrane potential. (C) Resting membrane potential. (D and E) Input resistance at RMP (D) and at −65 mV (E). The Kruskal-Wallis test was performed followed by Dunn’s multiple comparisons test. (F) Representative voltage responses to a depolarizing current step (150 pA; 750 ms) at RMR (G and H) Number of action potentials at RMP (G) and at −65 mV (H). The Two-Way ANOVA followed by Tukey’s post-hoc test was performed. * indicates a comparison between the control-Veh and the SPS-CORT group. ^#^ indicates a comparison between the control-CORT and the SPS-CORT group. ^$^ indicates a comparison between the SPS-Veh and the SPS-CORT group. Data are expressed as mean ± SEM. *P<0.05, **P<0.01, ***P<0.001, ****P<0.0001, ^#^P<0.05, ^##^P<0.01, ^###^P<0.001, ^$^P<0.05, and ^$ $^P<0.01. Further statistical information is provided in the Supplemental Information. Panel A was created with BioRender.com.

**Figure 4:**
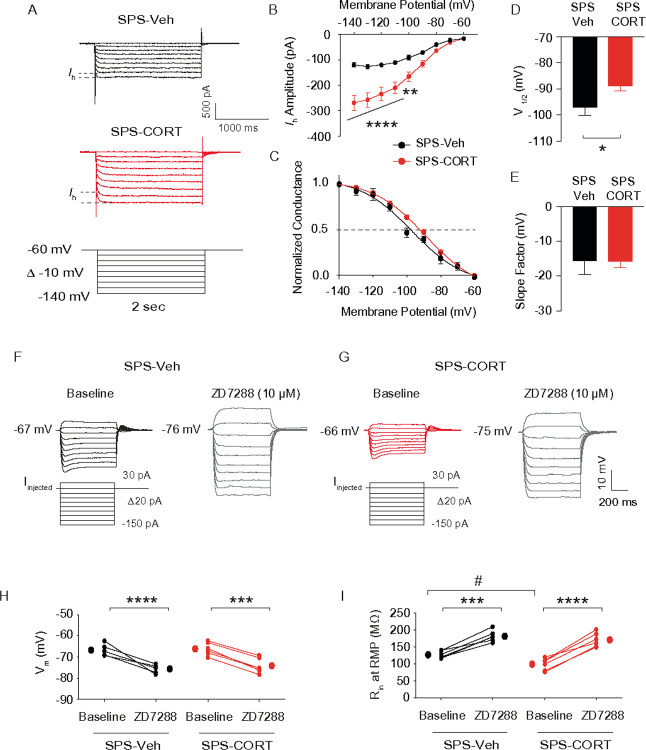
ZD7288 normalizes altered membrane properties in dorsal CA1 neurons of SPS-CORT mice. **(A)** Representative current responses to step voltage commands ranging from −140 mV to – 60 mV (Δ = 10 mV) at a holding potential of −60 mV in the dorsal CA1. The approximate position for determining the peak tail current is shown by gray vertical dashed lines. (B) *I*_*h*_ amplitude. The Two-Way ANOVA followed by Tukey’s post-hoc test was performed. (C) Voltage dependence of activation for *h* channel. The voltage dependence of activation (V_**1/2**_) for *h* channel was determined from tail currents (*I*_**h**_ / *I*_**h**_ max). The activation curve was fitted using a Boltzmann function, with the values of V_**1/2**_ and the slope factor. (D) V_**1/2**_ of *h* channel. The Mann-Whitney test was performed. (E) Slope factor. (F and G) Representative voltage responses to a current step (−150 pA to 30 pA, D10 pA, 700 ms) at resting membrane potential in SPS-Veh (F) and SPS-CORT (G) groups. (H) Resting membrane potential before and after bath application of ZD7288 (10 mM). The Wilcoxon matched-pairs signed rank test was performed separately for the SPS-Veh and SPS-CORT groups (I) Input resistance at RMP before and after bath application of ZD7288. The Wilcoxon matched-pairs signed rank test was performed separately for the SPS-Veh and SPS-CORT groups. The Mann-Whitney test was performed between SPS-Veh (baseline) and SPS-CORT (baseline) groups. Data are expressed as mean ± SEM. *P<0.05, **P<0.01, ***P<0.001, ****p<0.0001, and ^**#**^P<0.05. Further statistical information is provided in the Supplemental Information.

Given that SPS-CORT mice exhibited impairments in short-term spatial working memory, contextual fear recall, and fear extinction, we next investigated whether intrinsic membrane properties were altered in dorsal CA1 neurons. Using whole-cell current-clamp recordings, we assessed resting membrane potential (RMP), input resistance (R_in_), and action potential (AP) firing. Dorsal hippocampal slices were prepared following the 3-day extinction test (Fig. 3A). Compared to Control-Veh, Control-CORT, and SPS-Veh groups, SPS-CORT mice showed significantly reduced R_in_ at both RMP (Fig. 3D) and at a standardized membrane potential of – 65 mV (Fig. 3E), while RMP itself did not differ across groups (Fig. 3C). Moreover, AP firing was markedly decreased in the dorsal CA1 neurons of SPS-CORT mice at both RMP (Figs. 3F and 3G) and – 65 mV (Figs. 3F and 3H). These findings indicate a substantial reduction in the intrinsic excitability of dorsal CA1 neurons in SPS-CORT mice. Given previous reports of *I*_h_ enhancement following chronic stress paradigms such as chronic unpredictable stress^16^ and chronic social defeat stress^14, 17^ we next asked whether the reduced excitability observed in dorsal CA1 neurons of SPS-CORT mice might be associated with increased *I*_h_. To test this, we performed whole-cell voltage-clamp recordings to directly measure *I*_h_. Indeed, *I*_h_ was significantly elevated in dorsal CA1 neurons from SPS-CORT mice compared to SPS-Veh controls (Figs. 4A and 4B). Additionally, the voltage of half-maximal activation (V_1/2_) was shifted by approximately + 8 mV, indicating that HCN channels in these neurons were more likely to open at depolarized membrane potentials (Figs. 4C and 4D). The slope factor, however, remained unchanged (Figs. 4C and 4E), suggesting no alteration in channel cooperativity or gating steepness. Given the significant increase in *I*_h_ observed in the SPS-CORT group, we next sought to confirm whether this enhancement was mediated by HCN channels. To do so, we compared RMP and R_in_ at RMP before and after bath application of ZD7288 (10 μM), an HCN channel blocker, in SPS-Veh and SPS-CORT mice. ZD7288 significantly hyperpolarized the RMP in dorsal CA1 neurons from both groups, with no group difference observed post-treatment (Figs. 4F–4H). Consistent with our earlier findings of reduced R_in_ at RMP in SPS-CORT neurons (Fig. 3D), baseline R_in_ was significantly lower in the SPS-CORT group. However, following ZD7288 application, R_in_ values converged between groups (Figs. 4F,4G, and 4I), suggesting that the reduced excitability in SPS-CORT mice is primarily driven by enhanced HCN channel activity.

### HCN1 overexpression in SPS mice mimics behavioral impairments induced by post-CORT exposure.

**Figure 5:**
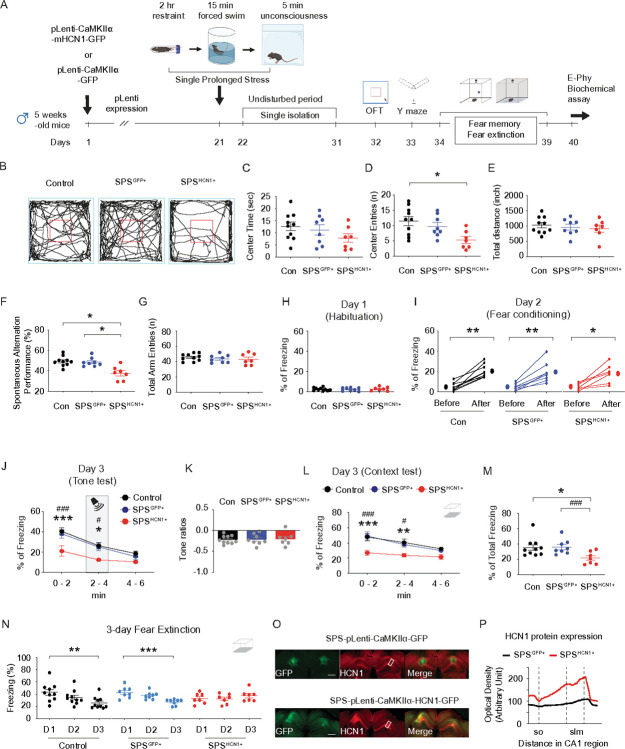
HCN1 overexpression in SPS mice induces PTSD-like behavioral deficits. (A) Schematic diagram of the experimental design. (B) Representative video tracking images of age-matched male mice during open filed test. (C) Center time. (D) Center entries. The Kruskal-Wallis test was performed followed by Dunn’s multiple comparisons test. (E) Total travelled distance. (F) Percentage of spontaneous alternation performance among groups. (G) Total arm entries. (H) Habituation (I) Fear acquisition on Day 2. The Wilcoxon matched-pairs signed rank test was performed separately for the control, SPS^GFP+^, and SPS^HCN1+^ groups. (J) Percentage of freezing during the tone memory test. The Two-Way ANOVA followed by Tukey’s post-hoc test was performed. * indicates a comparison between the control and the SPS^HCN1+^ group. ^#^ indicates a comparison between the SPS^GFP+^ and the SPS^HCN1+^ group. (K) Tone ratios. (L) Percentage of freezing during the context memory test. The Two-Way ANOVA followed by Tukey’s post-hoc test was performed. * indicates a comparison between the control and the SPS^HCN1+^ group. ^#^ indicates a comparison between the SPS^GFP+^ and the SPS^HCN1+^ group. (G) The total percentage of freezing during a 6-minute context memory test. The Kruskal-Wallis test was performed followed by Dunn’s multiple comparisons test. (N) Fear extinction during the 3-day fear extinction test. The Kruskal-Wallis test was performed followed by Dunn’s multiple comparisons test separately for the control, SPS^GFP+^, and the SPS^HCN1+^ group. (O) Representative coronal sections of the dorsal hippocampus showing the areas of the CA1 region infected by lentivirus and immunolabeled with an antibody against HCN1. The rectangle box depicts the region of the slice used for quantification of optical density. Scale bar 400 mm. (P) Quantification of HCN1 protein expression. Data are expressed as mean ± SEM. *P<0.05, **P<0.01, ***P<0.001, ^#^P<0.05, and ^###^P<0.001. Further statistical information is provided in the Supplemental Information. Panel A was created with BioRender.com.

**Figure 6:**
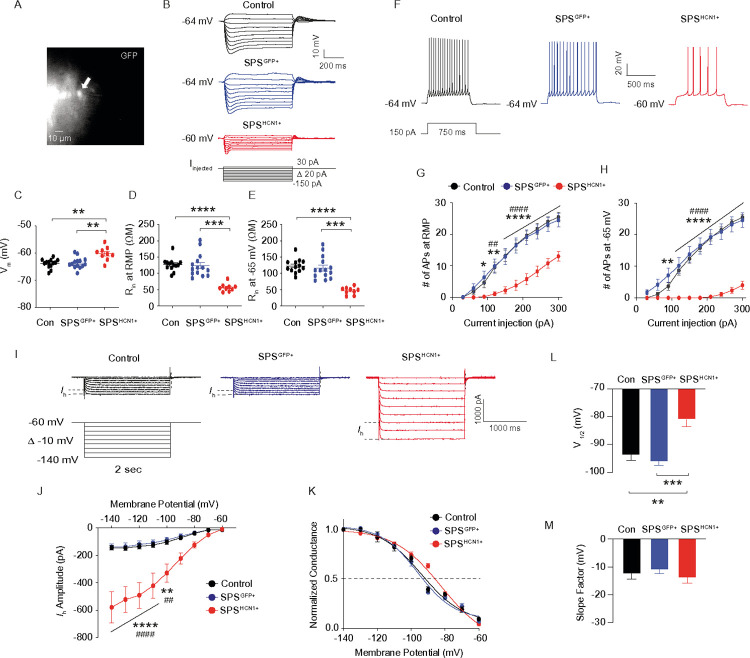
Reduced neuronal excitability and enhanced *I*_h_ in dorsal CA1 neurons of SPS^HCN1+^mice. (A) Photomicrograph of a representative recorded lentivirus-infected dorsal CA1 pyramidal neurons. The arrow indicates the recorded pyramidal neuron. (B) Representative voltage responses to a current step (−150 pA to 30 pA, D10 pA, 700 ms) at resting membrane potential. (C) Resting membrane potential. (D and E) Input resistance at RMP (D) and at −65 mV (E). (F) Representative voltage responses to a depolarizing current step (150 pA; 750 ms) at RMP. (G and H) Number of action potentials at RMP (G) and at −65 mV (H). (I) Representative current responses to step voltage commands ranging from −140 mV to – 60 mV (Δ = 10 mV) at a holding potential of −60 mV in the dorsal CA1. The approximate position for determining the peak tail current is shown by gray vertical dashed lines. (J) *I*_h_ amplitude. (K) Voltage dependence of activation for *h* channel. The voltage dependence of activation (V_1/2_) for *h* channel was determined from tail currents (*I*_h_ / *I*_h_ max). The activation curve was fitted using a Boltzmann function, with the values of V_1/2_ and the slope factor. (L) V_1/2_ of *h* channel. (M) Slope factor. The Kruskal-Wallis test was performed followed by Dunn’s multiple comparisons test in (C), (D), (E), and (L). The Two-Way ANOVA followed by Tukey’s post-hoc test was performed in (G), (H), and (J). * indicates a comparison between the control and the SPS^HCN1+^ group. ^#^ indicates a comparison between the SPS^GFP+^ and the SPS^hcn1+^ group. Data are expressed as mean ± SEM. *P<0.05, **P<0.01, ***P<0.001, ****P<0.0001, ^##^P<0.01, ^###^P<0.001, and ^####^P<0.0001. Further statistical information is provided in the Supplemental Information.

Because (1) SPS-Veh mice showed no behavioral deficits or alterations in intrinsic membrane properties,—alongside increased *I*_h_ and reduced neuronal excitability—we next asked whether overexpression of HCN1 alone could reproduce the behavioral phenotype observed in SPS-CORT mice. To address this, five-week-old male mice received lentiviral injections expressing either GFP (control) or HCN1 under the CaMKIIα promoter. After allowing three weeks for viral expression, mice were subjected to the SPS procedure. Following a 10-day undisturbed period, mice underwent a battery of behavioral tests to evaluate anxiety-like behavior, exploratory activity, spatial working memory, and fear-related memory. In the open field test, mice with HCN1 overexpression following SPS (SPS^HCN1+^) exhibited a significant reduction in center entries (Figs. 5B and 5D), while time spent in the center area remained unchanged (Figs. 5B and 5C). Total distance traveled did not differ across groups. Notably, although SPS^HCN1+^ mice entered the center less frequently, they tended to remain there longer, likely due to freezing-like behavior rather than exploratory motivation. In the Y-maze test, SPS^HCN1+^ mice exhibited a significantly lower spontaneous alternation performance (SAP) compared to both control and SPS^GFP^ mice groups (Fig. 5F), while total arm entries remained unchanged (Fig. 5G), indicating impaired spatial working memory without alterations in locomotor activity. We next evaluated fear-associated memory using contextual fear conditioning. No group differences were observed during habituation on Day 1 (Fig. 5H) or during fear acquisition on Day 2 (Fig. 5I). In the tone re-exposure test, SPS^HCN1+^ mice showed reduced freezing behavior (Fig. 5J), although tone discrimination ratios were comparable across groups (Fig. 5K), suggesting that tone alone did not drive the freezing response. Strikingly, SPS^HCN1+^ mice displayed impaired contextual fear recall, as evidenced by reduced freezing during context re-exposure (Fig. 5L), along with a significant decrease in total freezing time (Fig. 5M). Furthermore, these mice failed to exhibit normal extinction of the conditioned fear response over three days of context re-exposure (Fig. 5N), indicating deficits in fear extinction learning. Following behavioral testing, viral targeting of the dorsal CA1 and HCN1 overexpression were confirmed by immunohistochemistry (Figs. 5O and 5P). Following preparation of dorsal hippocampal slices, we performed whole-cell recordings from dorsal CA1 neurons expressing the viral construct (Fig. 6A). In current-clamp recordings, HCN1 overexpression resulted in membrane depolarization (Fig. 6C), along with a significant reduction in R_in_ both at RMP (Figs. 6B and 6D) and at – 65 mV (Figs. 6B and 6E). Action potential firing was also significantly reduced at both RMP (Figs. 6F and 6G) and – 65 mV (Figs. 6F and 6H), confirming diminished neuronal excitability. In voltage-clamp recordings, we observed a marked increase in *I*_h_ in HCN1-overexpressing neurons (Figs. 6I and 6J). Furthermore, the voltage of half-maximal activation (V_1/2_) was significantly depolarized (Figs. 6K and 6L), while the slope factor remained unchanged (Figs. 6K and 6M), indicating altered activation threshold without changes in gating kinetics.

## Discussion

This study identifies a critical interaction between stress exposure and glucocorticoid signaling that disrupts hippocampal function and behavior. Neither SPS-Veh nor Control-CORT groups exhibited significant changes in intrinsic membrane properties of dorsal CA1 neurons, including resting membrane potential, input resistance, or action potential firing. In contrast, dorsal CA1 neurons from SPS-CORT mice showed decreased input resistance, reduced neuronal excitability, and elevated *I*_h_, indicating a shift in intrinsic excitability that coincided with impairments in spatial working memory, contextual recall, and fear extinction, key features of PTSD-like behavior, despite normal anxiety-like behavior in the open field test. To probe the downstream mechanism, we overexpressed HCN1 selectively in dorsal CA1 neurons of SPS mice. This manipulation not only reproduced the cognitive deficits observed in SPS-CORT mice but also induced anxiety-like behavior, suggesting that elevated HCN1 activity contributes to both cognitive and emotional dysfunction, depending on its magnitude or anatomical context. Furthermore, electrophysiological recordings from SPS + HCN1 mice confirmed similar intrinsic alterations to those seen in the SPS-CORT group, including reduced input resistance and diminished firing output. Importantly, these physiological abnormalities in SPS-CORT mice were normalized by ZD7288, an HCN channel blocker, providing pharmacological evidence that HCN channel activity is central to this pathological state.

Our data show that SPS alone or corticosterone alone did not impair memory (Figs. 1 and 2) or alter intrinsic excitability in dorsal CA1 (Fig. 3), but their combination (SPS + CORT) triggered significant deficits. This indicates that prior stress primes hippocampal circuits, sensitizing them to subsequent glucocorticoid exposure - consistent with the “two-hit” model of PTSD, where an initial vulnerability (hit 1) must be followed by a significant challenge (hit 2) to precipitate pathology^21^. Such a mechanism may help explain why only a subset of trauma-exposed individuals develop PTSD, despite widespread HPA activation. In line with this framework, a modified SPS paradigm in which a second stressor—inescapable footshock—delivered after the SPS procedure enhances fear expression and anxiety-like behaviors in rats^25^, suggesting that secondary insults can exacerbate PTSD-like symptoms. Their findings further support the concept that initial stress exposure induces latent vulnerability, which can be unmasked by later stressors. Intriguingly, SPS effects differ in species. In male rats, SPS reliably impairs fear extinction and contextual memory^5–7^, while in male mice, the behavioral outcomes are often more variable and less consistent^8, 9, 26^ Although no direct comparisons of corticosterone kinetics or GR expression post-SPS exist, these species differences may reflect distinct HPA-axis dynamics and GR-associated sensitivity between rats and mice. Integrating these findings with our own, SPS + post-CORT model refines the “two-hit” framework by directly manipulating the endogenous stress hormone system, rather than relying on a secondary behavioral stressor. This hormone-centric approach allows us to precisely isolate the downstream effects of heightened glucocorticoid signaling on hippocampal circuitry and behavioral outcomes, offering a mechanistically distinct and translationally relevant model for stress-related disorders.

A growing body of evidence implicates maladaptive glucocorticoid receptor (GR) signaling as a core mechanism driving stress-related cognitive dysfunction, particularly within the hippocampus—a region critically involved in contextual memory and HPA axis regulation^27–31^. Studies using the SPS model consistently show that trauma exposure alters GR expression, phosphorylation, and downstream signaling^32–34^ Post-SPS corticosterone administration at 7 days after SPS leads to rapid upregulation of GR target gene expression in the dorsal hippocampus, indicating stress-induced hypersensitivity of GR signaling^35^. Blunted glucocorticoid responsiveness in a genetically selected rat line produces core PTSD-relevant traits—impaired fear extinction, smaller hippocampal volume, and disrupted REM sleep—highlighting GR dysfunction as a convergent vulnerability factor^36^. Building on this framework, we previously found that acute corticosterone treatment (within ~ 20 minutes) significantly increases HCN1 protein expression and membrane-bound *I*_h_ as measured by cell-attached recordings in dorsal CA1 pyramidal neurons^14^. This rapid effect is likely mediated by enhanced membrane trafficking via the auxiliary subunit tetratricopeptide repeat-containing Rab8b-interacting protein, as evidenced by increased membrane-bound *I*_h_^14^. These data suggest that glucocorticoids not only exert transcriptional control over HCN1 but can also rapidly alter its membrane availability and function through post-translational mechanisms, providing a direct link between acute stress hormone exposure and immediate changes in hippocampal excitability. In our current model, SPS followed by corticosterone administration (SPS-CORT) produces a sustained increase in *I*_h_ conductance, leading to reduced intrinsic excitability of dorsal CA1 neurons. This hypoexcitability is associated with behavioral deficits in working memory and contextual fear extinction—phenotypes that are absent in SPS-only or corticosterone-only conditions. Our findings suggest that upregulation of *I*_h_ serves as a maladaptive form of intrinsic plasticity that functionally decouples dorsal CA1 from afferent inputs (entorhinal cortex, CA3), thereby impairing memory consolidation^17^. Our results go further by showing that targeted overexpression of *HCN1* in dorsal CA1 neurons of SPS mice alone reproduced the same behavioral impairments observed in SPS mice infused with corticosterone. This phenocopy strongly supports a model where excessive GR activation acts through HCN1 upregulation to cause hippocampal dysfunction^14^. The mechanistic pathway aligns well with recent molecular evidence demonstrating that corticosterone, via GR and PKA, boosts TRIP8b, and HCN1 expression to downregulate neuronal excitability in dorsal CA1 neurons^14^. Moreover, Li et al. independently confirmed that glucocorticoids exert rapid, HCN channel–mediated suppression of CA1 pyramidal neuron activity^37^.

In conclusion, our study demonstrates that while SPS or corticosterone alone did not induce significant behavioral or physiological changes, their combination produced marked impairments in memory, contextual processing, and extinction learning, without affecting baseline anxiety. These deficits were associated with altered intrinsic excitability in dorsal CA1 neurons, including reduced input resistance and increased *I*_h_. *HCN1* overexpression in dorsal CA1 of SPS mice not only reproduced the cognitive deficits observed in the SPS + post-CORT group but also induced anxiety-like behaviors, suggesting that heightened HCN1 activity contributes to stress-induced behavioral impairments. While we did not directly manipulate glucocorticoid receptor signaling in this study, the phenocopy supports the idea that HCN1 upregulation acts downstream of stress hormone exposure. Together, these findings underscore the behavioral relevance of HCN1 dysregulation in the hippocampus and position it as a promising target for alleviating cognitive and emotional symptoms in stress-related disorders.

## Supplementary Files

This is a list of supplementary files associated with this preprint. Click to download.

- Supplementallnformation.docx
